# Clinical assessment of ^252^Californium neutron intracavitary brachytherapy using a two-channel Y applicator combined with external beam radiotherapy for endometrial cancer

**DOI:** 10.6061/clinics/2016(01)03

**Published:** 2016-01

**Authors:** Qian Zhou, Cheng Tang, Ke-Wei Zhao, Yan-Li Xiong, Shu Chen, Wen-Jing Xu, Xin Lei

**Affiliations:** Third Military Medical University, Cancer Center, Research Institute of Surgery and Daping Hospital, Chongqing, China

**Keywords:** Endometrial Cancer, Californium, Two-Channel Y Applicator

## Abstract

**OBJECTIVE:**

The aim of this study was to determine the efficacy of ^252^Californium neutron intracavitary brachytherapy using a two-channel Y applicator combined with external beam radiotherapy for the treatment of endometrial cancer.

**METHODS:**

Thirty-one patients with stage I–III endometrial cancer were recruited for this study. The stage I patients received only ^252^Californium neutron intracavitary brachytherapy with a two-channel applicator. The stage II and III patients received both ^252^Californium neutron intracavitary brachytherapy using a two-channel applicator and parallel-opposed whole pelvic radiotherapy.

**RESULTS:**

The five-year local control rate was 80.6% (25/31), the overall survival rate was 51.6% (16/31), and the disease-free survival rate was 54.8% (17/31). The incidence of serious late complications was 12.9% (4/31).

**CONCLUSIONS:**

^252^Californium neutron intracavitary brachytherapy using a two-channel applicator combined with external beam radiotherapy was effective for treating endometrial cancer and the incidence of serious late complications related to this combination was within an acceptable range.

## INTRODUCTION

Endometrial cancer is one of the most common gynecologic malignancies worldwide. According to a report from the American Cancer Society, approximately 52,630 new cases of endometrial cancer were diagnosed and 8,590 deaths were associated with endometrial cancer in the United States in 2014 1. The National Cancer Institute of China estimated that there would be 47,751 new cases of endometrial cancer in China in 2010 2. Currently, surgery remains the standard treatment for early-stage endometrial cancer. However, radiation has become a more suitable treatment option for patients with medically inoperable endometrial cancer or for those of advanced age.

In terms of a definitive radiotherapy for endometrial cancer, intracavitary brachytherapy (ICBT) alone can be used to treat stage I patients. Moreover, ICBT combined with external beam radiotherapy (EBRT) can be used to treat patients with local advanced-stage endometrial cancer. Generally, there are two types of brachytherapy using conventional γ-rays: high-dose-rate (HDR) brachytherapy using ^192^Ir or low-dose-rate (LDR) brachytherapy using^ 137^Cs. There have been no randomized controlled trials (RCT) comparing the effects of HDR brachytherapy and LDR brachytherapy on endometrial cancer in recent years because endometrial cancer is rarely treated with radiotherapy alone 3,4.

Many reports in the literature have described the use of californium-252 (^252^Cf) as a neutron source for the treatment of cervical cancer due to its higher relative biologic effectiveness (RBE) and lower oxygen enhancement rate (OER) than γ-rays 5,6. Moreover, the ^252^Cf neutron source has been used effectively for ICBT of endometrial cancer 7. However, a single-channel applicator was used in most endometrial cancer studies 8,9, including our own, which is described below 10. The use of a single-channel applicator has several drawbacks, including longer treatment durations and a slightly higher incidence of complications than the use of a two-channel applicator 9. Further, the dose distribution associated with a single-channel applicator is not ideal and is difficult to adjust. Between 1999 and 2004, our center performed ^252^Cf neutron ICBT using a single-channel applicator to treat 40 cases of endometrial cancer and we observed the above-described shortcomings associated with this method (i.e., long treatment durations, a high rate of complications and difficulty in adjusting the dose and the dose distribution).

The modification of two-channel (TC) Y (Rotte Y) applicators for brachytherapy has overcome many of the shortcomings associated with single-channel applicators, particularly with regard to adjustment of the optimal dose distribution in the craniocaudal and lateral directions. Therefore, TC applicators have become the standard fixed applicators used for γ-ray ICBT of endometrial cancer 11.

In 2007, our center began employing a ^252^Cf neutron source using the TC applicator for endometrial cancer treatment. The aim of this study was to assess the clinical outcomes of endometrial cancer patients treated via^ 252^Cf neutron ICBT using a TC applicator combined with EBRT.

## MATERIALS AND METHODS

### Subjects

Thirty-one stage I–III endometrial cancer patients diagnosed based on biopsy findings and staged according to the International Federation of Gynecology and Obstetrics (FIGO) guidelines from September 2007 to August 2011 were included in this study. Among these patients, the most common comorbidities associated with receiving radiotherapy alone included hypertension and diabetes (Table 1). None of the patients had received treatment before the study, and all endometrial cancer diagnoses had been confirmed by histology. All of the patients exhibited Karnofsky scores greater than 70 and hemoglobin levels greater than 90 g/L; their mean age was 55.9 years (range: 30–72 years) (Table 1).

All of the patients underwent a chest x-ray, an abdominal B-ultrasound, a pelvic CT or MRI, cystoscopy and a colonoscopy as components of their pre-treatment evaluation. During the course of treatment, a pelvic B-ultrasound and a gynecological examination were performed every week to examine changes in uterine size and uterine cavity depth.

### Treatment methods

#### Equipment

An LZH-1000 ^252^Cf neutron brachytherapy machine (Shenzhen Lingdun Technology, Shenzhen, China) and an 8 MV/X linear accelerator (Elekta Corp., Stockholm, Sweden) were used. For the 252Cf neutron source, the average energy was 2.3 MeV, with a half-life of 2.645 years; the shape was cylindrical with dimensions of 3 mm in diameter × 8 mm in height, an active region of 1.4 mm × 9.0 mm, and a source strength per unit volume of 500–200 μg. The following formula was used to calculate the 252Cf dose: equivalent biological dose (*DGY-eq*) = neutron relative biological effectiveness (RBE) of 252Cf × ^252^Cf neutron dose + γ-ray RBE x γ-ray dose; the 252Cf RBE ranged from between 4–6.

#### Treatment plan

**Figure 1-:**
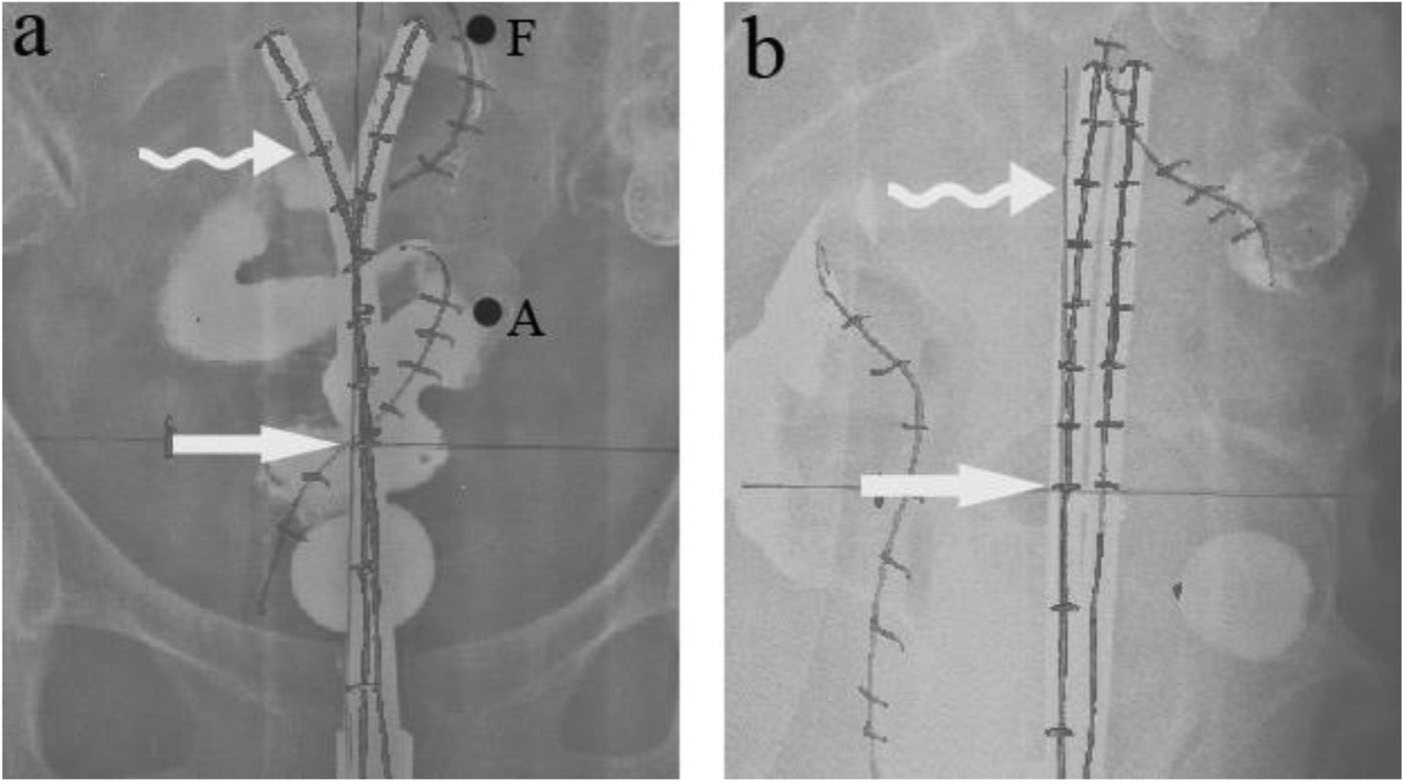
A) Antero-posterior view; B) anterolateral view. The anterolateral image was captured when the TC applicator was turned to the horizontal position; this image shows that the top of the two channels reached the two horns of the uterus. A and F denote dose reference points A and F, respectively. The cervix is indicated by arrows, and the uterus is indicated by curled arrows.

**Figure 2-:**
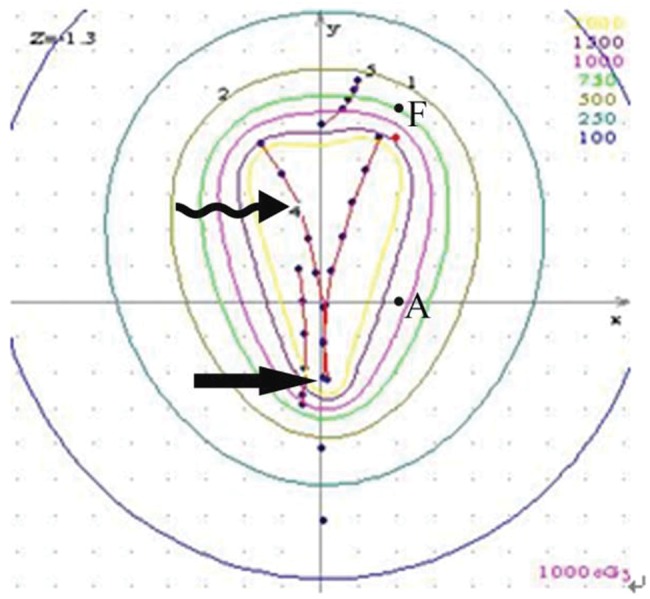
Isodose contour showing that the dose at point F was nearly identical to that at point A in stage II and III patients. A and F denote dose reference points A and F, respectively. The cervix is indicated by arrows and the uterus is indicated by curled arrows.

**Figure 3-:**
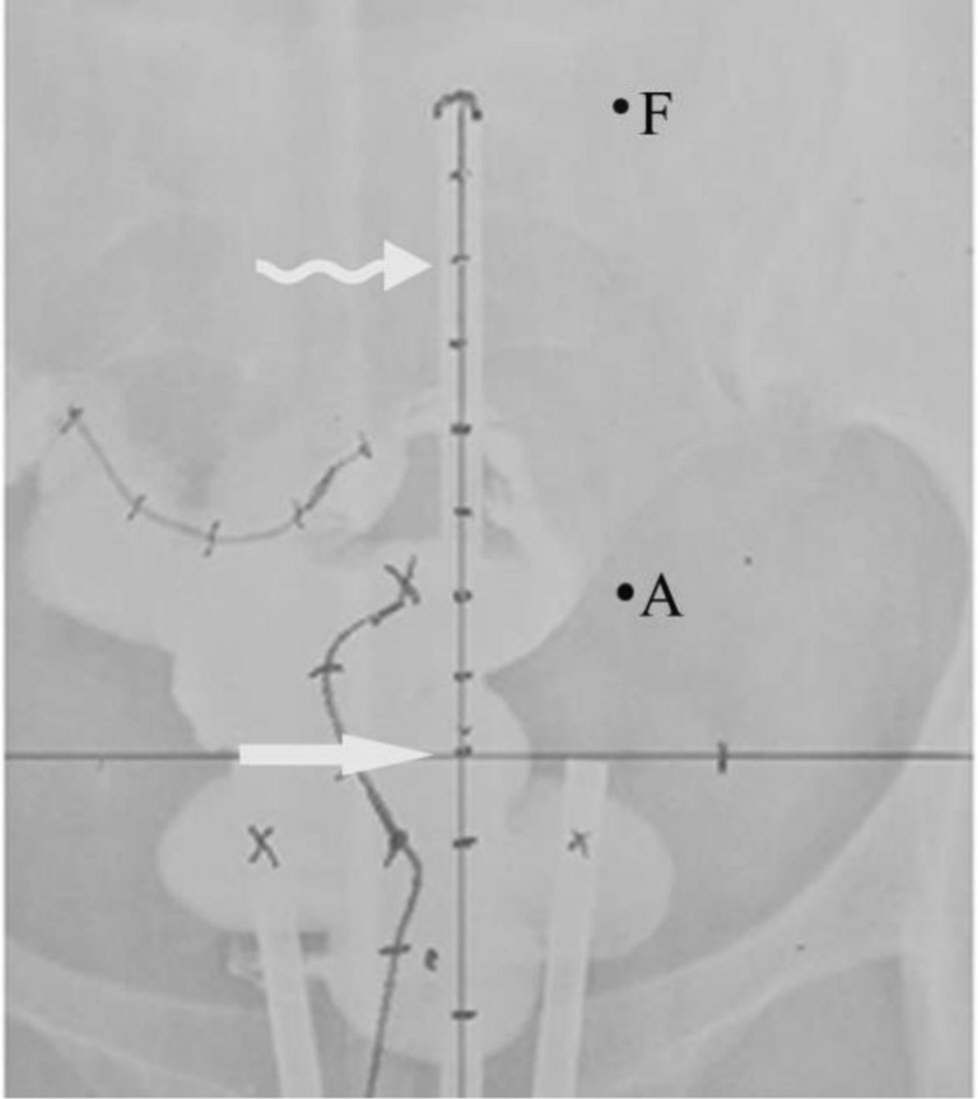
Image showing the method by which stage I and II endometrial cancer patients were administered a single channel and a vault tube of internal irradiation to supplement potentially insufficient doses at the uterine fundus and the vaginal fornix. A and F denote dose reference points A and F, respectively. The cervix is indicated by arrows, and the uterus is indicated by curled arrows.

**Figure 4-:**
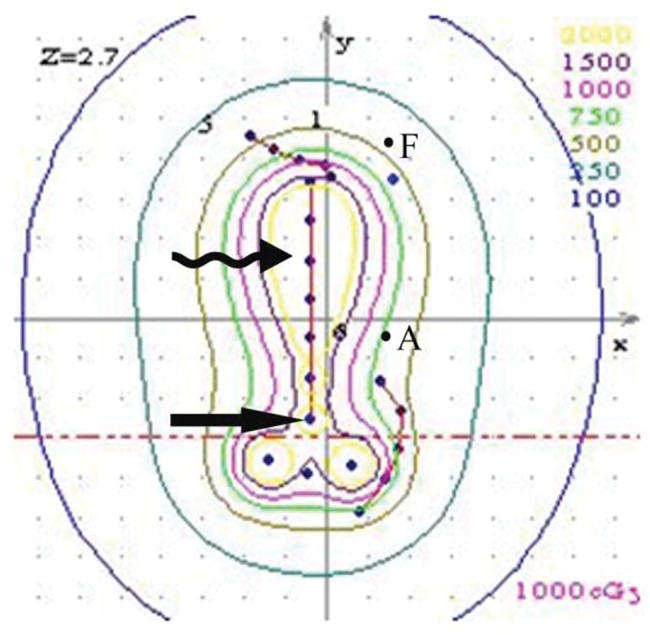
Isodose contour showing that the ratio of the dose applied to the cervix to the dose applied to the vaginal fornix was 1.0:(0.8–1.0) in stage I patients. A and F denote dose reference points A and F, respectively. The cervix is indicated by arrows, and the uterus is indicated by curled arrows.

**Figure 5-:**
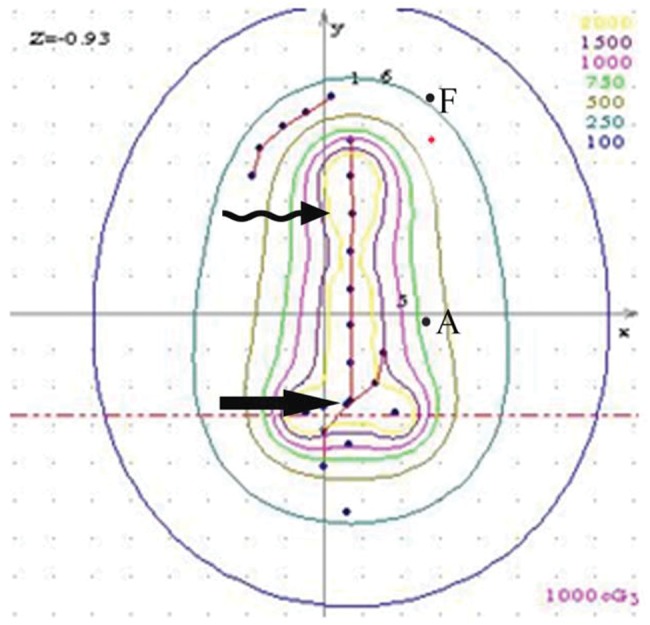
Isodose contour showing that the ratio of the dose applied to the cervix to the dose applied to the vaginal fornix was 1.0:(1.2–1.4) in stage II patients. A and F denote dose reference points A and F, respectively. The cervix is indicated by arrows, and the uterus is indicated by curled arrows.

Stage I patients underwent ^252^Cf neutron ICBT using the TC applicator alone. Stage II or III patients underwent _252_Cf neutron ICBT using the TC applicator combined with EBRT. All patients were hospitalized throughout the treatment period. External radiation was applied four times per week with opposing fields and a dose per fraction of 2 Gy. Once the total dose reached 20–36 Gy in 10–18 fractions, the central portion of the field was shielded using a 4 cm thick lead block, and the treatment was continued with antero-posterior field irradiation until the total dose reached 46–50 Gy. 252Cf neutron ICBT using the TC applicator was delivered after 4–5 fractions of EBRT. ICBT then continued once per week, with the EBRT applied on 4 other days of the week. The total treatment duration was 6-7 weeks. A non-steroidal painkiller or morphine was administered before applicator placement. Barium enemas and bladder catheterizations were prepared prior to injection of the contrast agent into the balloon. Then, between 7 and 12 cervical dilatation rods were used sequentially to gradually expand the cervix until it was sufficiently enlarged. The two channels of the applicator were turned to a vertical position such that the two front ends were closed together, followed by insertion of the fundus was inserted into the uterus. The TC applicator was then turned to the horizontal position such that the top of the two channels reached the two horns of the uterus, and an anterolateral image was captured (Figure 1). Points A and F (point F was defined on line A, 2 cm lateral to the top of the TC applicator) were chosen as the dose reference points. The brachytherapy dose was 40–45 Gy to point A and 48–55 Gy to point F in 4–5 fractions. For stage I patients, the dose at point F was higher than that at point A. For stage II or III patients, the dose at point F was approximately the same as that at point A (Figure 2). The dose at the posterior wall of the bladder and the rectum was adjusted based on the isodose contour so that the dose at the bladder and the rectum was below 80% of that at point A. Stage I or II patients were administered a single channel and a vault tube of internal irradiation (Figure 3) to supplement potentially insufficient doses at the uterine fundus and the vaginal fornix. The ratio of the dose applied to the cervix to the dose applied to the vaginal fornix was 1.0:(0.8–1.0) for stage I patients (Figure 4) and 1.0:(1.2–1.4) for stage II patients (Figure 5). During 252Cf neutron ICBT using the TC applicator, the patients were monitored for symptom alleviation, and gynecological examinations were performed weekly to determine the size of the uterus and the extent of tumor regression. At six months post-treatment, uterine curettage and histopathological examination were performed to examine tumor cell regression.

#### Follow-ups

Beginning one month post-treatment, a pelvic B-ultrasound was performed monthly. At three months post-treatment, uterine curettage and histopathological examination of the endometrial tissues were performed. Outpatient follow-up visits were required once every three months beginning three months post-treatment and once every six months beginning one year post-treatment. None of the patients were lost to follow-up.

#### Statistical analysis

SPSS13.0 software was used for statistical analyses. Discrete data are presented as frequencies (percentages); continuous data are presented as means ± SE. For statistical analyses, Chi-square contingency table analysis and Kaplan-Meier tests were performed; *p-*values<0.05 were considered statistically significant (Tables 2 and 3).

#### Ethics

This study was approved by the ethics committee of our hospital, and all patients signed informed consent forms.

## RESULTS

**Figure 6-:**
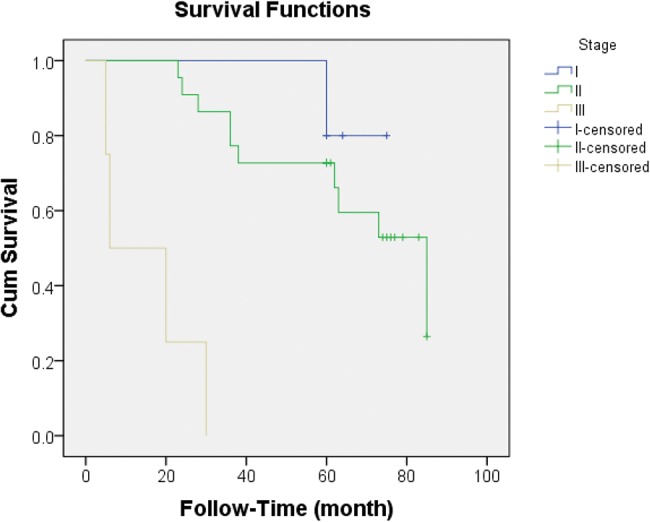
Survival curves of patients with different stages of endometrial cancer.

The mean follow-up duration was 54.8 months. Of the 31 patients enrolled in this study, 15 patients died during the follow-up period. One of the stage I patients died as a result of fatal coronary artery disease. Two stage III patients died from the persistent tumor. Three deceased patients had pelvic lymph node recurrence, four deceased patients had local recurrence at the time of death, and five deceased patients had distant metastases. The mean overall survival duration was 61.8±5.2 months, with a 95% confidence interval of 51.6–72.0 months (Table 3). The 5-year overall survival rate for all patients was 51.6% (Figure 6). The disease-specific survival (DSS) rates for stages I, II, and III were 100%, 54.5%, and 0%, respectively (Table 2).

Following treatment via ^252^Cf neutron ICBT using the TC applicator combined with EBRT, both pelvic B-ultrasound and gynecological examination showed that the uterus had shrunk substantially in all 31 patients and that the uterine cavity depth was reduced to a varying extent (0.5–5.0 cm). Of the two locally uncontrolled patients treated with an additional fraction of ^252^Cf neutron ICBT using the TC applicator, one experienced locally controlled disease, and the other, who showed clear cell histology, experienced local failure. The most common locations of disease recurrence were distant (16.1%), local recurrence alone (12.9%), and pelvic lymph nodes (9.6%). Of the five patients with distant tumor recurrence, three exhibited a single distant tumor involving the lungs and bone, and two exhibited multi-organ involvement. Additionally, patients with comorbid endometrial cancer and diabetes were more susceptible to metastasis development and disease recurrence (8/31) (Table 4).

All patients completed the treatment without interruption. All of the late complications were limited to grade 2, and there were no recto-vaginal or vesico-vaginal fistulas. Three patients had frequent urination, urgency, and hematuria at 26 months post-radiotherapy. Cystoscopic examination ruled out other diseases, and these patients were diagnosed with radiation cystitis. Another patient was diagnosed with radiation proctitis. The incidence of late complications was 12.9% (4/31) (Table 5).

## DISCUSSION

Surgery remains the primary standard treatment for patients with endometrial cancer. Definitive radiotherapy is typically offered to patients who suffer from medical comorbidities and those who have locally advanced tumors, as surgery may be not suitable for these patients. Therefore, patients treated via radiotherapy have a higher mortality rate than those treated via surgery. Nevertheless, radiotherapy remains the best option for patients with inoperable endometrial cancer. Thus, comparisons of survival between surgery and radiotherapy alone are of limited value. Patients with inoperable endometrial cancer are generally treated via ICBT alone or in combination with EBRT.

The most common histological type of endometrial cancer is adenocarcinoma, which is not as sensitive to conventional radiation as squamous cell carcinoma. Moreover, locally advanced stage endometrial tumors are often bulky and contain many hypoxic cells, which are resistant to conventional radiation. In contrast to conventional radioisotopes, the ^252^Cf neutron represents a high linear energy transfer (LET) ray with distinct radiation and biological characteristics. For example, the ^252^Cf neutron can cause greater direct damage to tumor cell DNA, leading to a higher rate of DNA double-strand breaks, than conventional photon radiation. The ^252^Cf neutron also has a higher RBE and a lower OER than photons 7. Thus, bulky endometrial adenocarcinoma cells may be particularly sensitive to ^252^Cf neutrons, and this form of radiotherapy could be used to treat endometrial adenocarcinoma patients.

However, ^252^Cf neutron ICBT has not been the main method of choice for endometrial cancer because the neutron source used for ICBT is large and expensive and because it is difficult to achieve precise biological equivalent dose calibration using conventional γ-rays. Thus, ^192^Ir and ^137^Cs ICBT are the most commonly used forms of ICBT worldwide, especially in Western Europe and North America. Moreover, the ^252^Cf neutron source used for ICBT was a neutron TC applicator that was larger than the γ-ray TC applicator. Dilatation of the cervix and the uterus is required to insert the TC applicator. In general, all of the endometrial cancer patients in our study tolerated TC applicator implantation well. Patients with late toxicity to radiotherapy in our study had grade 2 complications and were diagnosed with radiation proctitis or radiation cystitis.

Patients with locally advanced stage endometrial adenocarcinoma or with inoperable endometrial adenocarcinoma who are treated via a combination of γ-ray ICBT and EBRT often suffer from local-regional relapse. In the past two decades, the local control rate for patients with locally advanced stage endometrial adenocarcinoma has remained unsatisfactory, even when a salvage radical hysterectomy was performed. The local control rate was 76–84% for stage I–III endometrial adenocarcinoma patients treated via a combination of γ-ray ICBT and EBRT. Alternatively, ^252^Cf neutron ICBT has been found to be effective as a radiotherapy and to have radiobiological advantages for the treatment of radio-resistant advanced or bulky endometrial tumors 7. Our data showed that the local control rate for stage I–III endometrial adenocarcinoma patients was 80.6%. However, the local relapse rate of patients with stage I–II disease was 14.8% (4/27), and that of patients with stage III disease was 50%.

The selection of an applicator is also important for ICBT of endometrial adenocarcinoma. The TC “Y” applicator consists of two rigid applicators, which can reach the two uterine horns, permitting coverage of a greater uterine width than a single-channel applicator. The dose distribution of the TC applicator was more optimal than that of the single-channel applicator, such that a satisfactory local control rate might be more effectively achieved using the TC for ICBT of endometrial adenocarcinoma. However, few studies have examined the application of the “Y” applicator for treating endometrial cancer. Ohkubo et al. reported a satisfactory local control rate in ten patients with stage I–II endometrial cancer, none of whom suffered from local recurrence at five years 12. The same TC applicator was used by Coon et al. to treat 49 endometrial cancer patients (most of whom had stage I disease) via HDR ICBT; in that study, three patients (6.1%) experienced local failure and the median time to disease recurrence was 17 months. These two studies showing low rates of local recurrence further show that the radiation doses were sufficient for destroying the tumor in the uterus.

Differences in tumor stages and differential tumor grades are associated with distinct outcomes of endometrial cancer patients. Without question, higher tumor stage, lower differential tumor grade, and unfavorable tumor histological type are associated with poorer prognosis. Previous studies had shown that the five-year overall survival rates of stage I–III endometrial cancer patients treated via either ^137^Cs or ^192^Ir ICBT, with or without EBRT ^1-5^^9^,. In contrast, in the study by Maruyama et al., ^252^Cf neutron ICBT combined with EBRT and hysterectomy resulted in five-year overall survival rates of 83% for stage I, 37% for stage II, and 50% for stage III endometrial cancer patients 7. Our current data showed a five-year overall survival rate of 51.6%; this result is consistent with other results of LDR and HDR studies, although it is lower than that from the study by Maruyama et al. We speculate that the primary reason for the higher survival rate in the study by Maruyama et al. was that nearly half of the patients (14/31) in that study underwent a hysterectomy after receiving radiation therapy; the survival rate of 50% for stage III patients in that study was much higher than that in a previous report, which showed a survival rate of only 17.5% among those undergoing radiotherapy alone 17.

Treatment via ^252^Cf neutron ICBT combined with EBRT as performed in our study may be sufficient for patients with stage I–II endometrial cancer, as these patients showed a five-year overall survival rate of 59.3%. This survival rate is comparable to previous reports showing overall survival rates of 42–61% for stage I–II endometrial cancer patients 18-21. Our treatment outcomes for the four patients with stage III endometrial cancer showed an unsatisfactory mean survival duration of 15.3±6.0 months. Two stage III patients died with persistent tumors. Regarding the other two stage III patients, one died 20 months post-treatment due to lung metastasis, and the other exhibited pelvic lymph node recurrence at 30 months of follow-up. These findings suggest that alternative treatment strategies should be used for patients with stage III endometrial cancer.

In the course of using the “Y” applicator to treat endometrial cancer, two ovoids or a ring applicator and a single-channel applicator should be added to supplement the radiation dose to the vaginal vault; such a treatment generally increases the radiation dose to the bladder and the rectum. In fact, we observed late toxicity of grade 2 in 12.9% (4/31) of the patients; this frequency is similar to the results from most studies of stage I–III patients (Table 6). However, a study of 36 stage I patients by Nguyen et al. using one tandem and one ovoid or cylindrical applicator to perform HDR ICBT alone showed a frequency of late toxicity of up to 21% ^6^^22^.

Although much effort has been invested to identify more effective methods for ICBT of endometrial cancer, no completely satisfactory method has been established. Our retrospective study contains several limitations, including the accuracy of cancer diagnosis and staging, the socioeconomic status of the patients, advances in treatment, and nurse-led supportive care. In particular, we used 2-D treatment planning, which is not as effective as 3-D planning for reducing the dose to at-risk organs 23.

^252^Cf neutron ICBT using a TC applicator combined with EBRT is effective for the treatment of endometrial cancer, and the incidence of serious late complications related to this therapy is acceptable.

## AUTHORS CONTRIBUTION

Zhou Q participated in patients care, wrote the first draft of the manuscript and performed the literature review. Tang C, Lei X were responsible for the patient care, study design, draft of the manuscript and intellectual inputs. Zhao KW, Jia L, Shan JL, Xiong YL, Chen S, Xu Wen-Jing were responsible for data collection, data analysis and manuscript writing. All authors read and approved the final version of the manuscript.

## Figures and Tables

**Table 1:** Patient characteristics.

Characteristics	Number of patients (%)*
**Total number of patients**	31
**Age (y)**	
30–50	5 (16.0%)
51–60	18 (58.0%)
61–72	8 (26.0%)
Mean	55.9
**BMI (kg/m^2^)**	
Mean	27
Range	20–41
**Stage (FIGO)**	
I	5 (16.0%)
II	22 (71.0%)
III	4 (13.0%)
**Morphology**	
Adenocarcinoma	28 (90.0%)
Clear cell carcinoma	3 (10.0%)
**Histopathologic grade**	
1	11 (35.5%)
2	13 (41.9%)
3	5 (16.1%)
Unknown	2 (6.5%)
**Comorbid diseases**	
Hypertension	11 (35.5%)
Diabetes	11 (35.5%)
Coronary artery disease	3 (9.7%)
Systemic lupus erythematosus	2 (6.4%)
Others**	4 (12.9%)

**<?ENTCHAR ast?>:** Thirty-one patients were enrolled in the study.

**<?ENTCHAR ast?><?ENTCHAR ast?>:** Others included refusal of surgery or severe chronic obstructive pulmonary disease.

**Table 2:** Outcomes of endometrial cancer treated via ^252^Cf neutron intracavitary brachytherapy.

Stage	No.	LCR	OS	DSS	Late toxicity
**I**	5	100% (5/5)	80.0% (4/5)	100% (5/5)	0% (0/5)
**II**	22	81.8% (18/22)	54.5% (12/22)	54.5% (12/22)	18.2% (4/22)
**III**	4	50.0% (2/4)	0% (0/4)	0% (0/4)	0% (0/4)
**Total**	31	80.6% (25/31)	51.6% (16/31)	54.8% (17/31)	12.9% (4/31)
**X^2^**		3.026	5.510	8.496	0.961
*P*		0.066	0.037	0.004	0.378

No.: number of patients; LCR: local control rate; OS: overall survival; DSS: disease-specific survival.

**Table 3:** Overall survival duration of endometrial cancer patients receiving ^252^Cf neutron intracavitary brachytherapy.

FIGO stage	Mean±SE	95% CI	Median±SE	95% CI
**I**	72.0±2.7	(66.7,77.3)		
**II**	66.5±5.4	(56.0,77.0)	85.0±13.1	(59.4, 110.6)
**III**	15.3±6.0	(3.50, 27.0)	6.00±7.50	(0.00, 20.8)
**Total**	61.8±5.2	(51.6, 72.0	73.0±6.79	(59.9, 86.1)

SE: standard error; CI: confidential interval.

Mean overall survival duration=61.8±5.2 months (95% confidence interval, 5–85 months), X^2^=31.0, and *p*=0.000.

**Table 4:** The relationships of morphology and comorbid diseases with metastasis development and disease recurrence.

Site	Number	Morphology	Number	Comorbid diseases	Number
Local recurrence alone	4 (12.9%)	Adenocarcinoma	4 (12.9%)	Hypertension Diabetes	2 (6.4%) 2 (6.4%)
Pelvic lymph node	3 (9.6%)	Adenocarcinoma	3 (9.6%)	Diabetes COPD	2 (6.4%) 1 (3.2%)
Persistent	2 (6.4%)	Adenocarcinoma Clear cell carcinoma	1 (3.2%) 1 (3.2%)	Diabetes	2 (6.4%)
Distant	5 (16.1%)				
Lung	2 (6.4%)	Adenocarcinoma	2 (6.4%)	Hypertension Diabetes	1 (3.2%) 1 (3.2%)
Multi-organ	2 (6.4%)	Adenocarcinoma	2 (6.4%)	Hypertension SLE	1 (3.2%) 1 (3.2%)
Bone	1 (3.2%)	Adenocarcinoma	1 (3.2%)	Diabetes	1 (3.2%)

COPD: chronic obstructive pulmonary disease; SLE: systemic lupus erythematosus.

**Table 5:** Late toxicity.

Site	Number of grade II complications (%)
Cystitis	3 (9.6%)
Rectum	1 (3.2%)

**Table 6:** Literature review of the outcomes of inoperable stage I-IV endometrial cancer patients receiving radiotherapy.

Authors	Method	Number	Stage	Applicator	Local recurrence (%)	OS (%)	Late toxicity (%)
Inciura, et al. (16)	HDRB +/- EBRT	29	I–III	Three-channel	17.2 (4/29)	48.3 (5 y)	13.8 (4/29)
Rouanet, et al. (24)	LDRB +/- EBRT	147	I–III	—	24 (36/147)	58.4 (5 y)	11.7
Knocke, et al. (9)	HDRB +/- EBRT	280	I–III	One-channel	22.9 (64/280)	52.7 (5 y)	23.9
Churn and Jone (8)	HDRB +/- EBRT	37	I-II	One-channel	16 (6/37)	68.4 (5 y)	—
			III–IV						33 (5 y)
Current study	NBT +/- EBRT	31	I–III	Two-channel	19.4 (6/31)	51.6 (5 y)	12.9 (4/31)

OS: overall survival; LDRB: low-dose-rate brachytherapy; HDRB: high-dose-rate brachytherapy; EBRT: external beam radiation therapy; NBT: neutron brachytherapy.
